# Treatment of hypercholesterolaemia in older adults calls for a patient-centred approach

**DOI:** 10.1136/heartjnl-2019-315600

**Published:** 2019-11-28

**Authors:** Emma EF Kleipool, Johannes AN Dorresteijn, Yvo M Smulders, Frank LJ Visseren, Mike JL Peters, Majon Muller

**Affiliations:** 1 Internal medicine, Amsterdam UMC, Vrije Universiteit Amsterdam, Amsterdam, The Netherlands; 2 Vascular medicine, University Medical Centre Utrecht, Utrecht, The Netherlands

**Keywords:** lipid-lowering drugs, cardiovascular disease, older adults, frailty

## Abstract

Due to an increasing number of older adults with (risk factors for) cardiovascular disease (CVD), the sum of older adults eligible for lipid-lowering drugs will increase. This has risen questions about benefits and harms of lipid-lowering therapy in older adults with a varying number of (cardiovascular) comorbidities and functional status. The heterogeneity in physical and functional health increases with age, leading to a much wider variety in cardiovascular risk and life expectancy than in younger adults. We suggest treatment decisions on hypercholesterolaemia in adults aged ≥75 years should shift from a strictly 10-year cardiovascular risk-driven approach to a patient-centred and lifetime benefit-based approach. With this, estimated 10-year risk of CVD should be placed into the perspective of life expectancy. Moreover, frailty and safety concerns must be taken into account for a risk–benefit discussion between clinician and patient. Based on the Dutch addendum ‘Cardiovascular Risk Management in (frail) older adults’, our approach offers more detailed information on when not to initiate or deprescribe therapy than standard guidelines. Instead of using traditional risk estimating tools which tend to overestimate risk of CVD in older adults, use a competing risk adjusted, older adults-specific risk score (available at https://u-prevent.com). By filling in a patient’s (cardiovascular) health profile (eg, cholesterol, renal function), the tool estimates risk of CVD and models the effect of medication in terms of absolute risk reduction for an individual patient. Using this tool can guide doctors and patients in making shared decisions on initiating, continuing or deprescribing lipid-lowering therapy.

## Introduction

**Figure 1:**
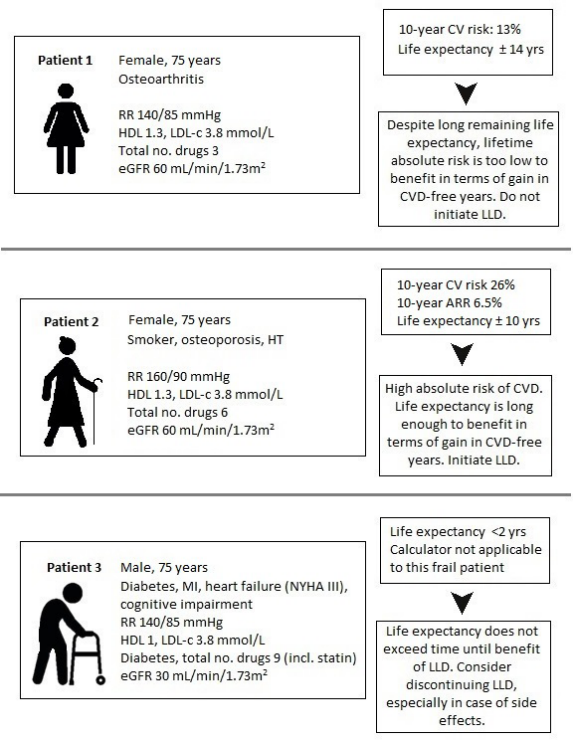
Cardiovascular risk profiles and potential treatment benefits of lipid-lowering therapy in patients 1 to 3. Estimations of pre-treatment risk of CVD and the potential treatment benefit of LLDs (ie, absolute risk reductions) are based on an older adult-specific, competing risk adjusted risk estimation tool.^12^ Estimated life expectancy is based on Holmes *et al*.^17^ Patients 1 and 2 have no pre-existing CVD. Except for hypertension (BP 160/90 mm Hg) and smoking, they have the same cardiovascular risk profile. Patient 1 receives vitamin D, calcium and acetaminophen and is otherwise in good health. Patient 2 receives calcium, vitamin D, a bisphosphonate, hydrochlorothiazide, amlodipine, macrogol and acetaminophen. She is in relatively good health. Both patients are currently not taking any lipid-lowering medication. Patient 3 has experienced a myocardial infarction 6 years ago, has heart failure (NYHA III), mild cognitive impairment, COPD and chronic renal failure (EGFR 30 mL/min/1.73 m^2^). He makes his way using a walker and uses nine drugs in total on a daily basis. He has been taking simvastatin 40 mg, without any evident side effects, once a day since his myocardial infarction. ARR, absolute risk reduction; BP, blood pressure; COPD, chronic pulmonary disease; CV, cardiovascular; CVD, cardiovascular disease; eGFR, estimated glomerular filtration rate; HDL, high-density lipoprotein; HT, hypertension; LDL-c, low-density lipoprotein cholesterol; LLD, lipid-lowering drug; MI, myocardial infarction; NYHA, New York Heart Association.

In Europe alone, at least 85 million patients have cardiovascular disease (CVD), causing 3.9 million deaths each year.^1^ The majority of these patients is aged ≥65 years, a number expected to grow even further in our ageing Western society. Hence, the sum of older adults eligible for lipid-lowering drugs will increase. This has given rise to clinical dilemmas on whether, and how we should treat hypercholesterolaemia in older adults, such as in patients 1, 2 and 3 presented in figure 1. All three cases present patients frequently encountered in daily clinical practice. They raise questions about the benefits and harms of lipid-lowering therapy in older adults with varying number of (cardiovascular) comorbidities and functional status. In this article we provide an overview of the current literature on lipid-lowering drugs in older adults and explain why heterogeneity of cardiovascular risk increases with age. We set out why and how estimated 10-year risk of CVD needs to be put into the perspective of a patient’s estimated life expectancy when deciding on whether or not to prescribe lipid-lowering therapy. Finally, we call for a shift from a risk-based strategy using traditional 10-year risk estimation tools to a lifetime benefit-based strategy, and provide recommendations on how to implement this in daily clinical practice.

## Evidence from trials

Up until now, only one randomised clinical trial (RCT), the PROspective Study of Pravastatin in the Elderly at Risk (PROSPER) trial,^2^ was specifically designed to evaluate statins versus placebo in older patients with and without pre-existing CVD. Other evidence on the benefits of lipid-lowering therapy in older adults is based on (meta-analyses of) subgroups of older patients from large RCTs including adults aged <80 years. The PROSPER trial^2^ included 5804 patients aged 70–82 with (high risk of) CVD. In older patients with CVD, pravastatin lowered the risk of cardiovascular events or death over a period of 3.2 years (HR: 0.78; 95% CI: 0.66 to 0.93). However, in patients without pre-existing CVD, contrary to most other primary prevention trials with effect sizes of 25%–30%,^3^ the relative risk (RR) reduction was only 6% and did not reach statistical significance (HR: 0.94; 0.77–1.15). This could be due to lack of power because the PROSPER trial was not designed to demonstrate a significant effect in each subgroup. Yet, this still leaves the effectiveness of statins in older adults without CVD unconfirmed. A recent meta-analysis of 28 randomised controlled trials including 14.483 patients >75 years of age (mean (SD) age 78.8 (2.8) years) showed that in patients with pre-existing CVD the proportional reduction in cardiovascular events was similar among all age groups (p_trend_ 0.2). The RR reduction on major vascular events for patients with CVD was 0.85; 95% CI 0.73 to 0.98 in 4.9 years of time.^4^ In patients without pre-existing CVD, mainly using subgroup results of the PROSPER trial, a smaller proportional risk reduction was observed with increasing age (p_trend_ 0.05). No significant benefit of statins was observed for patients aged >75 years without CVD (RR 0.92; 0.73 to 1.16). This is in line with a previously published primary prevention meta-analysis.^5^ Although ambiguous, of all older adults without CVD, data seem to suggest only (very) high-risk patients (eg, with diabetes) benefit from statins in terms of preventing CVD.^6–8^ In conclusion, previously performed trials show that lipid-lowering therapy benefits older adults at high absolute risk of CVD, for example, those with preexisting CVD. However, these trials did not include adults aged >80 years and most likely no frail older adults, suggesting that these results may not apply to a great deal of the (frail) older patients encountered in daily clinical practice. Also, RCTs were not powered in terms of outcomes which are important in the majority of older adults, such as quality of life and functional status. According to the National Institute on Aging (NIA) placebo-controlled and pragmatic RCTs seem to be suitable to address these knowledge gaps, studying a full range of outcomes, such as quality of life, function and symptom burden related to statins.^9^ However, we foresee obstacles for conducting such a trial, as today most older adults already take lipid- lowering drugs, and the frailest patients are not able to participate in such trials. Perhaps only pragmatic deprescribing RCTs or analyses of electronic health records with details on incidence and severity of major side effects and drug interactions could determine benefits and harms of (de)prescribing therapy in these older adults. Pending results of such trials, other factors should be taken into account when treating hypercholesterolaemia in the oldest old and frail older adults.

## Heterogeneity in cardiovascular risk and life expectancy

**Figure 2:**
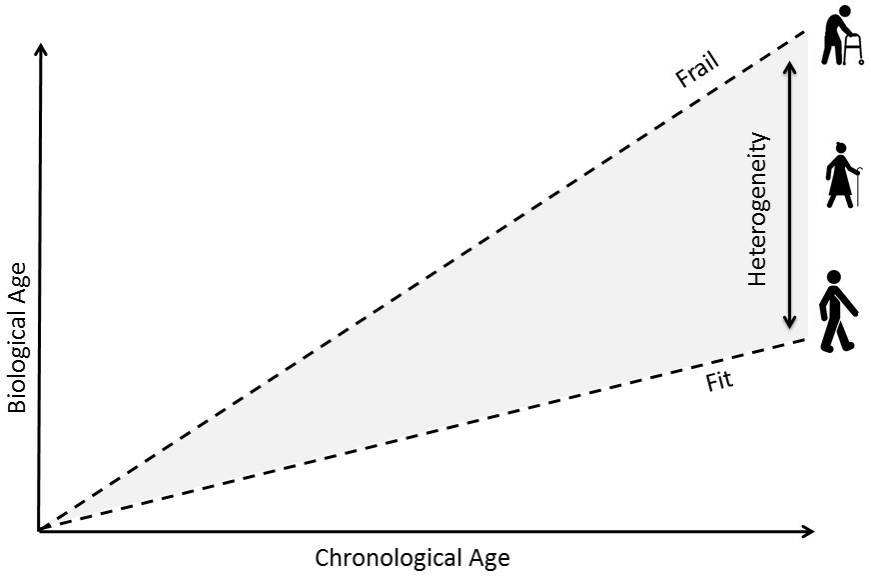
Increasing heterogeneity in biological age with increasing chronological age.

Older adults are likely to have multimorbidity, polypharmacy and functional impairments putting them at risk of frailty. This results in a heterogeneous population ranging from very fit patients with a long remaining life span to very frail older adults with a limited life expectancy (see online supplementary appendix 1 for more information on (assessing) frailty). With increasing chronological age, this difference in biological age becomes larger (figure 2). For every patient, potential benefit of lipid-lowering therapy depends on pre-treatment (ie, baseline) risk, the relative burden of treatable risk factors, for example, high blood pressure or cholesterol, and competing risks (ie, the risk of dying from other causes than CVD).^10 11^ Due to the large variety in cardiovascular risk factors, in older adults a much wider variety in baseline cardiovascular risk exists compared with younger adults.^12^ Differences in pre-treatment risk do not significantly influence the *RR* reduction established by lipid-lowering therapy,^13^ but pre-treatment cardiovascular risk does heavily influence the potential *absolute* risk reduction. For example, in two patients with the same estimated life expectancy, benefits of lipid-lowering therapy are greatest in the patient with the highest pre-treatment risk of CVD.^14 15^ However, it is important to realise that high pre-treatment risk of CVD does not necessarily mean a high lifetime risk of CVD.^14 15^ This can be explained by competing risks^16^; older adults are likely to have one or more chronic diseases, which puts them at high risk of non-vascular death.^17^ Consequently, they may not live long enough to experience a (new) cardiovascular event, making their 10-year risk of CVD lower than their lifetime risk of CVD (ie, the time-until-benefit exceeds life expectancy). As with cardiovascular risk, a substantial heterogeneity in life expectancy exists in older adults. For example, an average 75-year-old male has a median estimated remaining life expectancy of approximately 8–9 years.^18 19^ On the other hand, a 75-year-old with multiple comorbidities belonging to the ‘sickest’ 25% of the population is estimated to live a maximum of 5 years.^19^ See online supplementary appendix 2 for more information on estimating life expectancy.

10.1136/heartjnl-2019-315600.supp1 Supplementary data

## Rethinking 10-year risk of CVD

Table 1 gives an overview of the recommendations for managing hypercholesterolaemia in older adults for three major guidelines: the 2014 British National Institute for Health and Care Excellence (NICE) guideline,^20 21^ the 2016 European Society of Cardiology (ESC) guideline^22^ and the 2018 American Heart Association/American College of Cardiology (AHA/ACC) guideline.^23^ They all recommend that hypercholesterolaemia treatment in older adults, in principle, should not differ from those in younger patients, and is based on estimated 10-year cardiovascular risk. As previously experienced, CVD is a major risk factor for the occurrence of new cardiovascular events, all patients with pre-existing CVD are at very high 10-year risk of CVD and therefore eligible for lipid-lowering drugs. For patients without CVD, guidelines recommend estimating 10-year risk with traditional risk tools, which state that the majority of older adults are at (very) high 10-year risk of CVD because of their age (and presence of additional cardiovascular risk factors). As a consequence, almost every older adult—with or without CVD—is eligible for use of a lipid-lowering drug. Traditional risk estimation tools do not adjust for competing risks, and consequently, tend to overestimate an older patient’s actual 10-year risk of CVD. Also, as described above, a large number of older adults, especially those with multimorbidity or frailty, will not live as long as 10 years. On a group level, the beneficial effects of lipid-lowering drugs in terms of establishing a *clinically relevant difference* in preventing CVD occur approximately 2–3 years after initiating treatment.^2 24^ For some older patients, the time-until-benefit of lipid-lowering therapy therefore may exceed their life expectancy. Every guideline acknowledges that evidence on benefits and harms of lipid-lowering drugs in older patients, especially patients without CVD, is limited. They all mention that treatment decisions should be guided by clinical judgement, and recommend to include comorbidity, frailty, adverse effects, adherence and patient preference in the decision to treat or not. Compared to the 2014 NICE and the 2016 ESC guidelines, the 2018 AHA/ACC guideline clarifies in more detail what should be meant by this. Also, the latter guideline is the only guideline addressing when to consider deprescribing a lipid-lowering drug (although only for older patients without pre-existing CVD). An important shortcoming of all guidelines is that they are single-disease based, solely based on data from clinical trials in relatively young and fit adults and give advice on how to treat ‘average’ patients. Recently, a multidisciplinary addendum ‘Cardiovascular Risk Management (CVRM) in (frail) older adults’ was published in the Netherlands.^25^ Based on current, best-available evidence and expert opinion, it provides advice on starting, continuing or discontinuing lipid-lowering drugs in fit and frail older adults. The recommendations may be seen as progressive, because more attention is paid to frailty status, not initiating and deprescribing lipid-lowering therapy than in standard guidelines. Our recommendations presented below are based on this addendum.

**Table 1:** Guideline recommendations

Guideline	With CVD	Without CVD	Additional comments
NICE 2014 20 21	Treat in the same way as younger adults. Decisions on whether or not to start LLD therapy should be made after an informed physician–patient discussion about the risks and benefits of statin treatment	Treat in the same way as younger adults. Decisions on whether or not to start therapy should be made after an informed physician–patient discussion about the risks and benefits of LLD treatment	No/limited evidence exists to validate CV benefits and side effects of LLDs in oldest patients. Yet, the important effect of age on CV risk suggests all older people should be offered a LLD. Take benefits from lifestyle modifications, patient preference, comorbidities, polypharmacy, frailty and life expectancy into account
ESC 2016^22^	Treat in the same way as younger adults. However, recommendations should be followed with caution and common sense	Treat in the same way as younger adults. However, recommendations should be followed with caution and common sense	We encourage a discussion with patients regarding quality of life, life potentially gained, total burden of drug treatment and uncertainties of benefit. Monitor adverse effects closely, reconsider treatment periodically
AHA/ACC 2018^23^	70–75 years: treat in the same way as younger adults. >75 years: it is reasonable to initiate moderate/high intensity statins. Weigh potential CV risk reduction against adverse effects, drug–drug interactions, frailty and patient preferences before initiating therapy. Continue high-intensity statins if well-tolerated	70–75 years: treat in the same way as younger adults. >75 years: clinical assessment, risk discussion. It may be reasonable to stop statins when functional decline, multimorbidity, frailty or reduced life-expectancy limits the potential benefits of statins	

AHA/ACC, American Heart Association/American College of Cardiology; CV, cardiovascular; CVD, cardiovascular disease; ESC, European Society of Cardiology; LLD, lipid-lowering drug; NICE, National Institute for Health and Care Excellence.

## Making treatment decisions: from a risk-based to a benefit-based approach

Treatment decisions on hypercholesterolaemia in adults aged ≥75 years call for shift from a strictly 10-year cardiovascular risk-driven approach to a patient-centred and lifetime benefit-based approach in which shared decision-making is key. With the latter, estimated 10-year risk of CVD is also taking into consideration, but only after putting this into the perspective of life expectancy and by including potential negative effects of lipid-lowering therapy. To avoid overestimating an individual patient’s 10-year risk of CVD with traditional (older adults-specific) risk tools,^22 26 27^ we propose to use a competing risk adjusted, older adults-specific risk score.^12^ Contrary to traditional risk tools, this tool has been validated in studies that also included older patients and patients with several comorbidities.^12 28^ The tool is available free of charge on http://U-Prevent.com,^29^ and models the effect of medication changes in terms of 10-year absolute risk reduction (or number needed to treat). Together with the patient, risk is estimated after filling in easily available clinical parameters, such as cholesterol levels, blood pressure, renal function and number of drugs used. The clear graphics (figure 4, online supplementary appendix) can help facilitate a physician–patient discussion on whether the potential lifetime benefits versus the impact potential adverse effects are sufficient to validate therapy for the years left to live. This tool is less suitable in very frail patients, those with multimorbidity or those with a limited life expectancy, as these patients were not included in the (cohort) studies the tool algorithm is based on. Randomised studies on deprescribing lipid-lowering therapy in older adults are scarce, and there is no need to deprescribe therapy solely because of chronological age. However, we believe that there are two reasons to consider deprescribing lipid-lowering therapy: limited life expectancy (<1–2 years) and adverse effects overshadowing preventive treatment benefits. In patients with a limited lifespan, lifetime risk of CVD has fallen to such a degree that it is unlikely treatment will prevent (new) cardiovascular events, even in patients at (very) high baseline risk of CVD. In these patients, quality of life, functional status and doing no harm take priority over preventing (fatal) CVD.^23 30–32^ Although observational studies observed increased risk of cardiovascular events after deprescribing statins in older patients,^33^ randomised deprescribing trials observed no increase in mortality after deprescribing statins, and a potential to improve quality of life.^30 34^

## Adverse effects of lipid-lowering therapy

Common concerns on lipid-lowering therapy in older adults are statin-related muscle symptoms (eg, myalgia, weakness) leading to physical inabilities, drug–drug interactions and polypharmacy. ‘Real-life’ observational studies suggest a higher prevalence of statin-related muscle symptoms compared with those reported in RCTs (8%–11% vs 1%–5%).^35 36^ And although data suggest that statins are similarly tolerated in adults aged ≥75 years and younger adults,^37^ this may not be the case on individual patient level; preexisting functional limitations and polypharmacy could make older patients more prone to the muscle symptoms, functional decline and drug–drug interactions.^31 32 38 39^ These harmful effects may disproportionally affect frail older adults because they have an increased likelihood of poor functional status to begin with.^40^ Clear evidence for this is lacking.^38^ However, considering the large number of older adults taking statins, the likelihood of under-reporting (non-specific) muscle symptoms and their potential to majorly impact functionality, it is important to pay attention to adverse effects in every older patient.

## Practical implications

**Figure 3:**
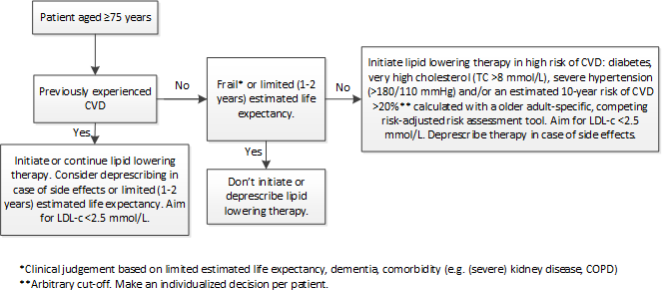
Flowchart on treatment of hypercholesterolaemia with lipid-lowering drugs in patients aged ≥75 years. The recommendations are based on the Dutch addendum ‘CVRM in (frail) older adults’.^25^ COPD, chronic obstructive pulmonary disease; CVD, cardiovascular disease; CVRM, cardiovascular risk management; LDL-C, low-density lipoprotein cholesterol; TC, total cholesterol.

For use in daily clinical practice, we constructed a flowchart (figure 3) with advice on when to initiate, continue and deprescribe lipid-lowering drugs in fit and frail adults aged ≥75 years. The recommendations are in line with the Dutch addendum ‘CVRM in (frail) older adults’.^25^ The cut-off of ≥75 years of age is arbitrary, but consistent with the majority of available evidence. For each individual patient, a discussion of estimated lifetime risk of CVD, risk of adverse effects and consideration of patient preferences should precede the decisions on prescribing lipid-lowering drugs. In older patients with pre-existing CVD with a reasonable life expectancy (more than 1–2 years), lifetime risk of CVD is considered high enough to benefit from initiating or continuing lipid- lowering therapy. However, in case of (severe) negative effects (eg, side effects) or if life expectancy is limited (less than 1–2 years), the benefits of lipid-lowering therapy do not outweigh potential adverse effects of treatment. For frail older patients without pre-existing CVD we propose to deprescribe or not to initiate lipid-lowering treatment. Their lifetime risk of CVD is not sufficient enough to benefit from lipid-lowering therapy in terms of preventing a cardiovascular event in the time left to live. Also, frailty may exacerbate adverse effects of therapy. In fit older adults with a life expectancy of ≥2 years, only initiate or continue lipid-lowering therapy if a patient is at high risk of CVD; diabetes mellitus, total cholesterol >8 mmol/L, blood pressure >180/110 mm Hg and/or a 10-year risk of CVD estimated with an competing risk adjusted, older adults-specific risk tool >20%. In low-risk patients without CVD, lifetime risk of CVD is too low to meaningfully benefit from therapy. At the same time, therapy potentially exposes these patients to adverse effects and promotes polypharmacy.

## Recommendations for patients 1–3

The following treatment recommendations are based on the Dutch addendum ‘CVRM in (frail) older adults’ (see flowchart presented in figure 3). Except for hypertension and smoking status, patient 2 has the same cardiovascular risk profile as patient 1 (figure 1). This puts patient 2 at higher risk of CVD. This is not reflected in estimated life expectancy (±10 years in patient 2 versus ±14 years in patient 1), but in 10-year risk of CVD (26% in patient 2 vs 13% in patient 1). Because the 10-year risk of CVD is <20% in patient 1, the benefit of lipid-lowering therapy in terms of lifetime absolute risk reduction or gain in CVD-free years would be considered too low to initiate lipid-lowering therapy by most clinicians and patients. Of course, although the >20% risk is commonly used in guidelines, the threshold for starting treatment is arbitrary. Per patient, an individualised decision must be made. Although patient 2 is expected to live less long than patient 1 (±14 vs ±10 years), her 10-year risk of CVD of 26% puts her at high lifetime risk of CVD. Benefit of lipid-lowering therapy is therefore greater in patient 2 compared with patient 1 in terms of lifetime absolute risk reduction and gain in CVD-free survival. Thus, it may be reasonable to initiate a lipid-lowering drug in patient 2, while the benefits in patient 1 do not outweigh potential negative effects of therapy. Patient 3 is currently taking simvastatin 40 mg daily. Despite his high absolute risk of a new cardiovascular event, it may be reasonable to deprescribe his statin, especially in case of side effects. Due to his comorbidities and limited level of cognitive and physical functioning, it is likely that patient 3 will die a non-vascular death within 1–2 years. Benefit of his statin in terms of preventing CVD is thus not very likely. Potential muscle-related side effects of his statin may have a disproportionally negative effect on his physical abilities as he has a poor physical status to start with. As similar patients (eg, limited life expectancy (1–2 years)) are under-represented in the (cohort) studies the competing risk adjusted, older adults-specific risk tool is based on, we do not recommend estimating cardiovascular risk with this tool for this frail patient.

## Conclusion

In the older population, a wider distribution of cardiovascular risk exists compared with younger adults due to heterogeneity in physical and functional health. Treatment decisions on hypercholesterolaemia in adults aged ≥75 years should call for shift from a strictly 10-year cardiovascular risk-driven approach to a patient-centred and lifetime benefit-based approach by putting estimated 10-year risk of CVD into the perspective of remaining life expectancy, patients preferences, frailty and potential adverse effects of treatment. When estimating 10-year risk of CVD, use a competing risk adjusted, older adults-specific risk score instead of traditional risk tools as the latter tend to overestimate risk of CVD. Evidence on the benefits versus harms of lipid-lowering therapy is inconclusive and inconsistent for a large number of older adults, particularly adults aged >80 years and frail older adults. We therefore advocate for randomised (de)prescribing trials studying a full range of outcomes, such as quality of life, functional capacity and symptom burden related to statins.
