# Supplementary material for: Treatment of hypercholesterolaemia in older adults calls for a patient-centred approach
Source: Heart. 2019 Nov 28;106(4):261–6. doi: 10.1136/heartjnl-2019-315600 (PMC7027025; doi:10.1136/heartjnl-2019-315600)
Supplement: Supplementary data [file heartjnl-2019-315600supp001.pdf]

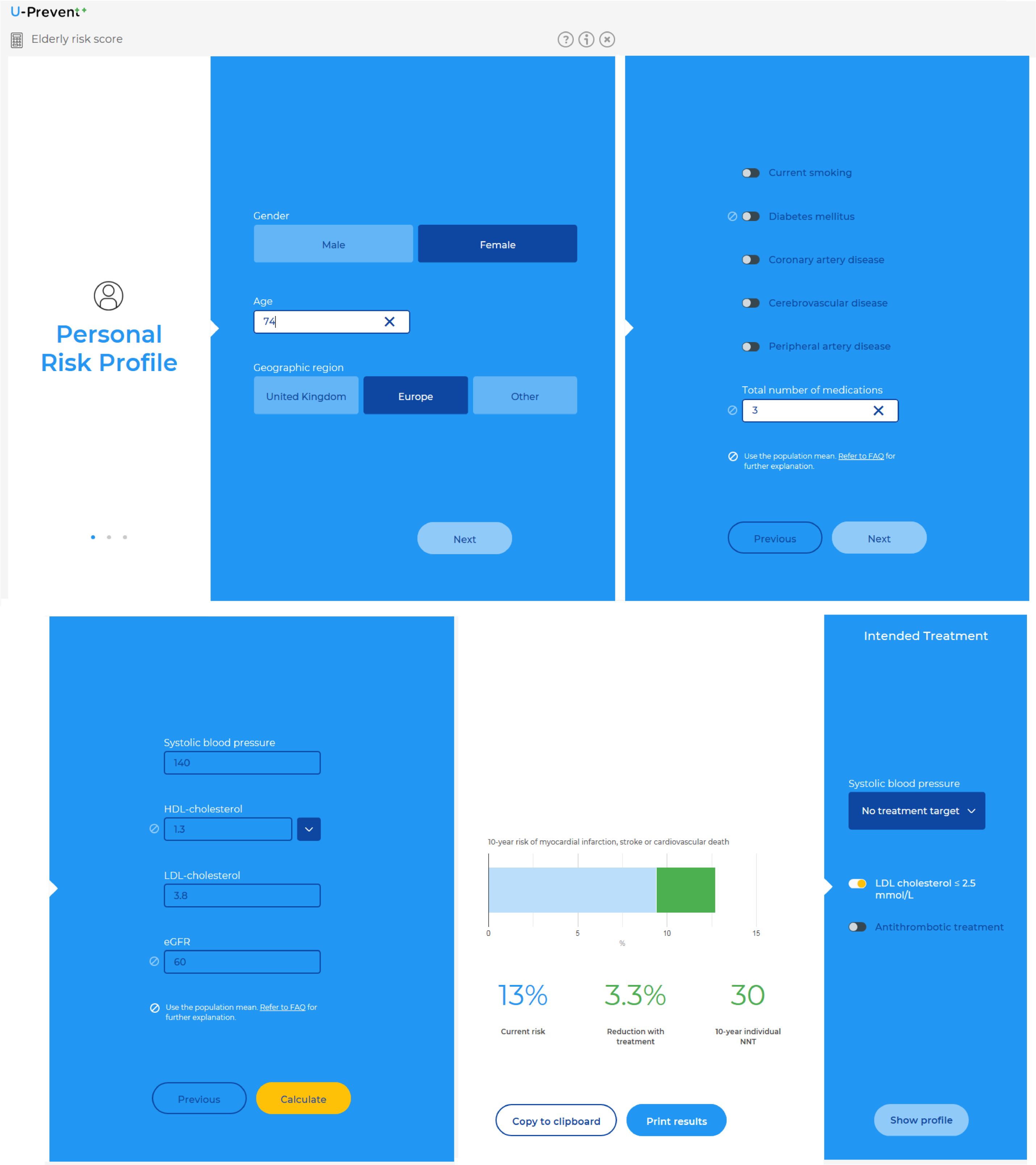
U-Prevent
Elderly risk score
Personal Risk Profile
Gender
Male
Female
Age
74
Geographic region
United Kingdom
Europe
Other
Next
Current smoking
Diabetes mellitus
Coronary artery disease
Cerebrovascular disease
Peripheral artery disease
Total number of medications
3
Use the population mean. Refer to FAQ for further explanation.
Previous
Next
Systolic blood pressure
140
HDL-cholesterol
1.3
LDL-cholesterol
3.8
eGFR
60
Use the population mean. Refer to FAQ for further explanation.
Previous
Calculate
10-year risk of myocardial infarction, stroke or cardiovascular death
0
5
10
15
%
13%
Current risk
3.3%
Reduction with treatment
30
10-year individual NNT
Copy to clipboard
Print results
Intended Treatment
Systolic blood pressure
No treatment target
LDL cholesterol ≤ 2.5 mmol/L
Antithrombotic treatment
Show profile
